# Dual pathway inhibition with faricimab for previously treated neovascular age-related macular degeneration and diabetic macular oedema: guidance from a UK panel of retina specialists

**DOI:** 10.1038/s41433-024-03223-w

**Published:** 2024-08-30

**Authors:** Louise Downey, Sobha Sivaprasad, Ramandeep Chhabra, Clare Bailey, Soma Chakrabarti, Samer Elsherbiny, Jignesh Patel, Giuliana Silvestri, Sarah-Lucie Watson, Gwyn Williams, Antony Parker, Saima Khokhar, Andrew Lotery

**Affiliations:** 1 Hull University Teaching Hospitals NHS Trust, Hull, UK; 2 NIHR Moorfields Clinical Research Facility, Moorfields Eye Hospital NHS Foundation Trust, London, UK; 3 Manchester Royal Eye Hospital, Manchester University NHS Foundation Trust and Faculty of Biology, Medicine and Health, University of Manchester, Manchester, UK; 4 University Hospitals Bristol and Weston NHS Foundation Trust, Bristol, UK; 5 NHS Greater Glasgow & Clyde, Glasgow, UK; 6 South Warwickshire University NHS Foundation Trust, Warwick, UK; 7 Colchester District General Hospital, Colchester, UK; 8 Belfast Health and Social Care Trust, Belfast, UK; 9 Royal Berkshire NHS Foundation Trust, Reading, UK; 10 Swansea Bay University Health Board, Singleton Hospital, Swansea, UK; 11 Roche Products Limited, Welwyn Garden City, UK; 12 Southampton Eye Unit and Faculty of Medicine, University Hospital Southampton NHS Foundation Trust, University of Southampton, Southampton, UK

**Keywords:** Retinal diseases, Physical examination

## Abstract

**Background/objectives:**

Some eyes with neovascular age-related macular degeneration (nAMD) and centre-involving diabetic macular oedema (DMO) fail to respond sufficiently or lose response over time to standard of care intravitreal anti-vascular endothelial growth factor (anti-VEGF) therapy. This paper explores clinical scenarios for switching to dual action angiopoietin-2 (Ang-2)/VEGF-A inhibitor faricimab (Vabysmo, Roche Products Limited) in previously anti-VEGF-treated patients.

**Methods:**

A national steering group meeting of UK retina specialists was held in London on 27 October 2023. Clinician practice and experience were reviewed together with pivotal clinical trial data and early findings from real-world settings. Roche Products Limited facilitated and funded the meeting.

**Results:**

While there is no standardised protocol for identifying suboptimal response, the authors review relevant clinical biomarkers of disease activity used in routine clinical practice to determine patient response and guide treatment decisions. Common reasons identified for considering a change of treatment were lack of efficacy demonstrated by suboptimal anatomic or visual improvement and insufficient durability of response. The panel outline strategies for switching to faricimab among eligible patients with a prior anti-VEGF treatment history, with initial monthly loading doses or maintaining the previous treatment interval before attempting to extend, that may be integrated into current treat-and-extend (T&E) clinical pathways for treating patients with nAMD and DMO. General considerations for switching between treatments are also reviewed.

**Conclusion:**

Clinicians may consider a treatment switch to faricimab in nAMD and DMO patients who have suboptimal disease control or insufficient durability of response on initial anti-VEGF therapy.

## Introduction

Standard of care for the treatment of neovascular age-related macular degeneration (nAMD) and visual impairment due to centre-involving diabetic macular oedema (DMO) is intravitreal anti-vascular endothelial growth factor (anti-VEGF) therapy [1, 2]. Anti-VEGF treatment offers significant visual and anatomic benefits in most patients with nAMD and DMO [1–3]. However, frequent anti-VEGF injections are often needed to maintain efficacy and some eyes fail to respond, only partially respond, or lose response over time and require review to assess alternative or additional treatment [4–6].

Faricimab (Vabysmo, Roche Products Limited) was approved in the United Kingdom (UK) in 2022 for the treatment of nAMD and DMO [7, 8], providing an additional effective treatment option—beyond VEGF inhibition alone—for both treatment-naïve and previously anti-VEGF-treated patients. Faricimab is a bispecific antibody that acts through inhibition of two distinct pathways by neutralisation of both angiopoietin-2 (Ang-2) and VEGF-A [7]. Through dual pathway inhibition, faricimab is designed to reduce vascular permeability and inflammation, inhibit pathological angiogenesis and restore vascular stability [9, 10].

Ang-2 and VEGF-A are considered key drivers of vascular leakage, neovascularisation and inflammation in retinal vascular diseases [11]. Ang-2 competitively inhibits binding of Ang-1 to Tie2 tyrosine kinase receptor, counteracting vascular homoeostasis maintained through Ang-1–dependent Tie2 activation [9]. Ang-2 inhibition in combination with VEGF inhibition may promote vascular stability beyond anti-VEGF therapy alone [9–12]. Levels of Ang-2 have been shown to be increased in nAMD and DMO and dual Ang-2 and VEGF-A inhibition blockade may provide added clinical benefit over anti-VEGF monotherapy [9, 12–14].

Contemporary guidance on switching strategies among anti-VEGF-experienced patients with nAMD and DMO is limited. The aim of this paper is to provide clinician-led practical guidance for switching to faricimab for suboptimal response to previous intravitreal anti-VEGF treatment for nAMD and DMO. The guidance is primarily intended to inform clinicians as well as allied healthcare professionals involved in treating and monitoring patients with nAMD and DMO in UK clinical practice. Application of panel recommendations is at the discretion of the treating clinician.

## Methods

A national steering group meeting of 11 UK medical retina specialists and two medical affairs partners from Roche Products Limited was held in London on 27 October 2023, focused primarily on treatment strategies for nAMD and DMO patients who have suboptimal response to previous anti-VEGF therapy. Roche Products Limited facilitated and funded the steering group meeting, and participants from Roche Products Limited provided presentations on faricimab clinical trial designs and outcomes. Following a review of pivotal faricimab clinical trial data and evidence from post hoc exploratory analyses, the panel considered current clinical practice, experience and opinion with respect to:

- clinical biomarkers and effectiveness outcomes relevant to assessment of patient response to anti-VEGF treatment; and
- strategies for switching to faricimab as an additional option for patients with suboptimal improvement on initial anti-VEGF treatment.

The meeting was followed by author reviews of a draft summary report; all authors contributed to subsequent development and finalisation of the approved manuscript.

## Pivotal clinical trial data and exploratory analyses

**Fig. 1:**
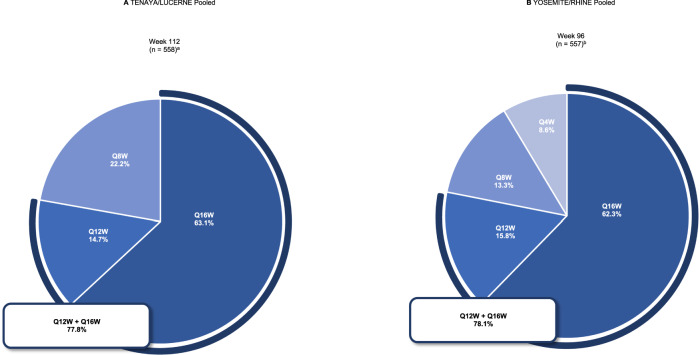
Extended dosing with faricimab in the pivotal nAMD and DMO trials. Faricimab T&E dosing intervals achieved at 2 years in TENAYA/LUCERNE pooled (**A**) and YOSEMITE/RHINE pooled (**B**). ^a^Percentages are based on the number of patients randomised to the pooled faricimab arms on Q8W, Q12W or Q16W dosing at week 112, among those who had not discontinued the study at that visit. Treatment interval at a given visit is defined as the treatment interval decision followed at that visit. Interval at week 112 is calculated using data recorded at week 108. ^b^Percentages are based on the number of patients in the pooled faricimab T&E arms on Q4W, Q8W, Q12W or Q16W dosing at week 96, among those who had not discontinued the study at the week 96 visit. Dark blue lines indicate the proportion of faricimab-treated patients on 12-week or 16-week dosing intervals. Q4W every 4 weeks, Q8W every 8 weeks, Q12W every 12 weeks, Q16W every 16 weeks, T&E treat-and-extend. Adapted from Khanani et al. [17].

The clinical efficacy, durability of response (frequency of injection) and safety of intravitreal faricimab in nAMD and DMO have been demonstrated in the global phase III TENAYA/LUCERNE (NCT03823287/NCT03823300) and YOSEMITE/RHINE (NCT03622580/NCT03622593) trials, respectively [7, 15–18]. Across the four trials, ~80% of patients treated with faricimab treat-and-extend (T&E) (previously referred to as personalised treatment interval) achieved 12-week or 16-week dosing intervals at 2 years [7, 17, 18] (Fig. 1). Treatment with faricimab was well tolerated through study ends, with a safety profile comparable to aflibercept (Eylea, Bayer) [7, 17, 18] (Table 1).

**Table 1:** Pooled safety data through study end in TENAYA/LUCERNE and YOSEMITE/RHINE.

AEs Through Study End, Patients With ≥1 AE, *n* (%)^a^	Pooled TENAYA/LUCERNE (nAMD)		Pooled YOSEMITE/RHINE (DMO)		
	Faricimab T&E *n* = 664	Aflibercept Q8W *n* = 662	Faricimab Q8W *n* = 630	Faricimab T&E *n* = 632	Aflibercept Q8W *n* = 625
Ocular AEs^b^	358 (53.9%)	345 (52.1%)	313 (49.7%)	311 (49.2%)	284 (45.4%)
Serious ocular AEs^b^	29 (4.4%)	29 (4.4%)	26 (4.1%)	34 (5.4%)	20 (3.2%)
Ocular AEs of special interest^c^	40 (6.0%)	43 (6.5%)	25 (4.0%)	33 (5.2%)	20 (3.2%)
Intraocular inflammation events^d^	20 (3.0%)	15 (2.3%)	9 (1.4%)	11 (1.7%)	7 (1.1%)
Uveitis	4 (0.6%)	3 (0.5%)	3 (0.5%)	4 (0.6%)	0
Iritis	8 (1.2%)	3 (0.5%)	1 (0.2%)	4 (0.6%)	2 (0.3%)
Iridocyclitis	2 (0.3%)	1 (0.2%)	2 (0.3%)	3 (0.5%)	1 (0.2%)
Vitritis	4 (0.6%)	1 (0.2%)	2 (0.3%)	0	2 (0.3%)
Post-procedural inflammation	0	5 (0.8%)	1 (0.2%)	1 (0.2%)	2 (0.3%)
Chorioretinitis	1 (0.2%)	0	0	1 (0.2%)	0
Keratic precipitates	2 (0.3%)	0	0	1 (0.2%)	0
Non-infectious endophthalmitis	0	1 (0.2%)	0	0	0
Keratouveitis	0	0	0	1 (0.2%)	0
Anterior chamber flare	0	1 (0.2%)	0	0	0
Endophthalmitis events	3 (0.5%)	2 (0.3%)	2 (0.3%)	4 (0.6%)	1 (0.2%)
Retinal vasculitis events	0	0	0	0	0
Retinal occlusive events					
Retinal vein occlusion	0	0	1 (0.2%)	4 (0.6%)	0
Retinal artery occlusion	0	0	1 (0.2%)	2 (0.3%)	2 (0.3%)
Retinal artery embolism	1 (0.2%)^f^	0	0	0	1 (0.2%)
Serious non-ocular AEs	138 (20.8%)	162 (24.5%)	175 (27.8%)	161 (25.5%)	173 (27.7%)
APTC events^e^	22 (3.3%)	20 (3.0%)	34 (5.4%)	30 (4.7%)	32 (5.1%)

Adapted from Khanani et al. [17].

*AE* adverse event, *APTC* Antiplatelet Trialists’ Collaboration, *BCVA* best-corrected visual acuity, *Q8W* every 8 weeks, *Q16W* every 16 weeks, *T&E* treat-and-extend.

^a^Results are presented for the pooled safety-evaluable populations. Percentages are based on *n* values in the column headings; multiple occurrences of the same AE in an individual are counted only once.

^b^Ocular AEs in the study eye only are presented.

^c^Ocular AEs of special interest were defined as events associated with severe intraocular inflammation, events requiring surgical or medical intervention to prevent permanent loss of sight or events associated with BCVA loss of ≥30 letters for >1 h.

^d^Excluding endophthalmitis.

^e^APTC events were adjudicated by an external independent committee; all other events were investigator reported.

^f^Hollenhorst plaque that was reported at the end of year 1 and was not treatment related as per the investigator.

In post hoc exploratory evaluations of pivotal faricimab clinical trials in nAMD and DMO, findings favour faricimab over aflibercept across multiple anatomic biomarkers for disease control, including absence of retinal fluid, absence of DMO, resolution of macular leakage, secondary epiretinal membrane (ERM) formation and resolution of retinal hyperreflective foci (HRF) [17, 19–25] (Table 2).

**Table 2:** Post hoc exploratory analyses of pivotal phase III trials of faricimab in nAMD and DMO.

nAMD: Pooled post hoc analyses of TENAYA and LUCERNE		
Biomarker	Results	Conclusion
CST reduction and absence of retinal fluid [19]	•Mean (95% CI) CST change from baseline at week 12: −145.4 µm (−149.1, −141.8) with faricimab (*n* = 665) vs. −133.0 µm (−136.7, −129.3) with aflibercept (*n* = 664) •Proportion without IRF and SRF at week 12 was 77% with faricimab vs. 67% with aflibercept	Greater anatomic improvements with faricimab compared with aflibercept during the matched monthly dosing period through week 12
Resolution of IRF and SRF [19]	•Time to first absence of IRF and SRF from baseline for the 75th percentile was week 8 (2 injections) with faricimab and week 12 (3 injections) with aflibercept	Time to first absence of IRF and SRF from baseline for the 75th percentile was 4 weeks sooner and with fewer injections with faricimab vs. aflibercept Q8W
**DMO: Pooled post hoc analyses of YOSEMITE and RHINE**		
Absence of DMO (CST < 325 µm) [17]	•Time to first absence of DMO for the 75th percentile was week 20 with faricimab Q8W (*n* = 632) (5 injections) and T&E (*n* = 632) (4 injections) vs. week 36 with aflibercept Q8W (*n* = 627) (7 injections)	Faricimab-treated patients (both Q8W and T&E) achieved first absence of DMO faster (16 weeks earlier) and with fewer injections vs. aflibercept Q8W
Absence of IRF [20]	•Time to first absence of IRF from baseline for the 50th percentile was week 48 for faricimab-treated patients (7 and 9 injections for faricimab T&E and Q8W, respectively) vs. week 88 for aflibercept-treated patients (13 injections)	Time to first absence of IRF from baseline for the 50th percentile was 40 weeks sooner for faricimab-treated patients compared with aflibercept-treated patients
Resolution of macular leakage [21]	•At week 16 following matched monthly dosing: •Median macular leakage area was 3.6 mm^2^ with faricimab (Q8W and T&E, *n* = 1128) vs. 7.6 mm^2^ with aflibercept Q8W (*n* = 560) •Proportion with resolution of macular leakage: 28.2% vs. 15.2%, respectively	Following matched monthly dosing, faricimab-treated patients (Q8W and T&E) achieved a greater reduction from baseline in macular leakage area on FFA and a greater proportion demonstrated resolution of fluorescein macular leakage, compared with those receiving aflibercept
Secondary ERM formation [22]	•Cumulative incidence of ERM formation through week 100 was 3.8% (23/602), 5.1% (31/608) and 7.6% (45/590) with faricimab Q8W, T&E and aflibercept Q8W, respectively	Through 2 years, the cumulative incidence of ERM formation was lower with faricimab vs. aflibercept
Resolution of retinal HRF [23, 24]	•In the inner retina 1 mm diameter: •The adjusted mean HRF volume reductions from baseline to week 48 were −118.29 pL with faricimab Q8W, −130.05 pL with faricimab T&E and −58.67 pL with aflibercept Q8W •For time to absence of HRF count at 2 consecutive visits, the 25th percentile was reached at approximately week 36 with faricimab Q8W and T&E compared with approximately week 44 with aflibercept Q8W	Findings show greater and faster resolution of HRF through week 48 in faricimab-treated patients compared with aflibercept-treated patients

*nAMD* neovascular age-related macular degeneration, *CI* confidence interval, *CST* central subfield thickness, *DMO* diabetic macular oedema, *ERM* epiretinal membrane, *FFA* fundus fluorescein angiography, *HRF* hyperreflective foci, *IRF* intraretinal fluid, *OCT* optical coherence tomography, *Q8W* every 8 weeks, *SRF* subretinal fluid, *T&E* treat-and-extend.

## Switching guidance for suboptimal anti-VEGF response in nAMD

### Clinical biomarkers

Significant optical coherence tomography (OCT) imaging biomarkers associated with nAMD activity and progression include intraretinal fluid (IRF), subretinal fluid (SRF), pigment epithelial detachment (PED), subretinal hyperreflective material (SHRM), new macular haemorrhage and HRF [26]. Intraretinal fluid is considered the most relevant in terms of impact (progression or improvement) on visual outcome [26]. In eyes treated with anti-VEGF for nAMD, persistent IRF is associated with worse long-term visual acuity compared with resolved IRF, while early resolution of SRF is associated with a greater improvement in visual acuity [26–28].

Signs of nAMD disease activity that guide retreatment decisions include: new or increased retinal fluid (IRF, SRF or subretinal pigment epithelium fluid) on OCT; increased PED size; new or persistent macular haemorrhage; new or increased SHRM; or decreased visual acuity of ≥5 Early Treatment Diabetic Retinopathy Study (ETDRS) letters attributable to choroidal neovascularisation activity [29, 30]. Fluorescein leakage or increase in lesion size on FFA may also indicate active disease [31]. Guidance from the National Institute for Health and Care Excellence (NICE) underscores the importance of monitoring of both eyes when one eye only is being treated for nAMD [32].

Clinicians mostly treat nAMD using a flexible T&E dosing regimen after an initial monthly treatment phase (i.e. loading) [7, 29, 33]. Treatment interval extension is considered appropriate when there is no retinal fluid or other OCT biomarkers of active nAMD, with stable or better visual acuity or if visual acuity loss is not considered due to nAMD disease activity [33]. Visual acuity measurements within high throughput real-world services may not provide an accurate assessment of disease activity status.

### Determining treatment response

A patient whose visual acuity declines due to persistent exudative disease activity despite an optimally delivered treatment regimen is considered a non-responder [34]. The original diagnosis should be re-evaluated, and the treating clinician should assess protocol adherence to rule out undertreatment [34]. A suboptimal response to anti-VEGF treatment may be considered as presence of macular fluid after the initiation phase of consecutive monthly anti-VEGF injections or persistent/residual macular fluid at any time post the initial loading phase [35]. Mettu et al. defined incomplete response to anti-VEGF therapy in nAMD as persistent disease activity (characterised by persistent fluid exudation, unresolved or new haemorrhage and progressive lesion fibrosis) and/or suboptimal visual recovery (failure to achieve visual acuity of ≥70 ETDRS letters or ≥20/40 Snellen equivalent) despite sustained treatment [36]. Refractory nAMD is generally considered based on the presence of persistent or recurrent IRF or SRF on OCT despite maximal (monthly) or prolonged anti-VEGF dosing for ~12 months or longer after treatment initiation [37].

### Options for switching from initial anti-VEGF to faricimab in nAMD

Current practice in treating nAMD is to actively consider switch alternatives for non-response and suboptimal response to previous anti-VEGF therapy at any time after the initial treatment loading phase and for high-need patients being treated regularly every 4–7 weeks (Q4W–Q7W, ~7–12 injections each year). A change may also be considered for treatment-resistant or recalcitrant nAMD, e.g. worsening visual outcomes, or persistent or recurrent retinal fluid on OCT, despite continued timely anti-VEGF injections over a 12-month or longer period [38]. Patients maintained on every-8-week (Q8W) anti-VEGF treatment (~6 injections each year), who cannot be extended further and patients who are not able to maintain intervals longer than 10 or 12 weeks (~4–5 injections per year), may be offered or request the option of a switch to potentially longer-acting treatment, if suitable.

Switching decisions may either be disease activity driven (anatomic control supplemented by visual acuity response) or based on insufficient durability of response per clinician and patient decision. Local service models, disease characteristics and patient-related factors including likely visit adherence may impact clinician decision-making on alternative effective treatment options.

For patients with suboptimal improvement after initial monthly loading and for patients with persistent retinal fluid or other signs of active disease on continued Q4W–Q7W dosing intervals, clinicians should consider initiating the treatment switch to faricimab with a loading phase of four consecutive monthly injections, if possible. Among patients previously treated with anti-VEGF at maximum every-4-week (Q4W) intervals, initial loading with monthly faricimab injections may continue to improve anatomic response.

**Fig. 2:**
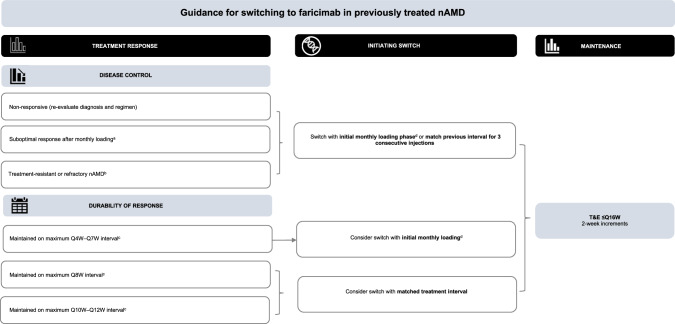
Guidance for switching to faricimab in previously treated nAMD. ^a^Suboptimal response defined as intraretinal fluid or subretinal fluid on OCT, other anatomic features of active or worsening disease (e.g. new haemorrhage OR new SHRM), or unchanged (≤5-letter improvement)/reduced VA due to nAMD, after three consecutive monthly intravitreal injections. ^b^Treatment-resistant nAMD generally defined as persistent retinal fluid on OCT despite continued intravitreal anti-VEGF injections over a 12-month period. ^c^Primarily maintained at this treatment interval or later shortened. ^d^In patients previously anti-VEGF-treated at maximum Q4W intervals, initial loading with monthly injections may continue to improve anatomic features. nAMD neovascular age-related macular degeneration, OCT optical coherence tomography, Q4W–Q7W every 4–7 weeks, Q8W every 8 weeks, Q10W–Q12W every 10–12 weeks, Q16W every 16 weeks, SHRM subretinal hyperreflective material, T&E treat-and-extend, VA visual acuity.

Among patients considered unable to extend beyond Q8W treatment intervals, a treatment switch to faricimab may be initiated by matching the previous interval, or with initial monthly injections per clinician discretion, followed thereafter by flexible dosing using a T&E regimen. Patients who are interval matched but who fail to respond sufficiently on first clinic review post switch may benefit from a new initiation phase of consecutive monthly injections. Figure 2 provides an overview of guidance for switching to faricimab T&E in previously treated nAMD.

## Switching guidance for suboptimal anti-VEGF response in DMO

### Clinical biomarkers

Early and sustained control of central subfield thickness (CST) and fluid resolution are both important for optimising vision outcomes in DMO [39, 40]. Additional biomarkers associated with treatment response in DMO include intraretinal cysts, large outer nuclear layer cyst, disorganisation of retinal inner layers (DRIL), ellipsoid zone disruption and amount and location of HRF [41].

Retreatment decisions in the management of DMO are based mainly on comparison of OCT features and visual acuity over recent visits. Change in OCT CST/central retinal thickness (CRT) should be assessed against the lowest CST/CRT achieved and visual acuity change against the best-achieved score in response to treatment, which usually occur following the monthly treatment phase but may be following further intensive treatment [42]. Extension of treatment interval using a personalised T&E regimen is considered appropriate once CST/CRT on OCT and visual acuity are stable [42].

### Determining treatment response

Non-response to treatment for DMO may be considered where there is worsening visual outcomes, or unchanged/increased CST/CRT, despite an optimally delivered treatment regimen. As for nAMD, diagnosis should be re-evaluated for eyes that are non-responsive to initial intravitreal treatment. A suboptimal clinical response at ≥12 weeks from treatment initiation may be considered where there is <20% reduction from reference OCT CST/CRT (machine matched for comparisons) and unchanged (≤5-letter improvement) or reduced visual acuity due to DMO with residual disease activity. The panel suggested a practical threshold for defining insufficient anatomic response as a reduction of less than 20% in reference CST/CRT (documented for example at time of diagnosis) and/or retinal thickness above an indicative absolute CST/CRT value or threshold as determined by the treating clinician (e.g. 390 µm if NICE guidance recommendation of CRT criterion of ≥400 µm was used to initiate anti-VEGF treatment) [43]. Treatment-resistant or refractory DMO is generally considered as persistent DMO, increased retinal thickness, or reduced visual acuity attributable to DMO, despite continued anti-VEGF treatment over a 12-month or longer period [44].

### Options for switching from initial anti-VEGF to faricimab in DMO

Clinicians may consider switching to faricimab in patients with DMO who are either non-responsive or considered suboptimal responders after monthly loading with anti-VEGF monotherapy, and for patients with persistent active disease following regular anti-VEGF treatment post loading. Other options for these patients include intravitreal corticosteroid implant treatment or supplemental focal laser.

In DMO, clinical management is focused predominantly on achieving effective disease control by continuing to treat until resolution of macular oedema, with an early switch considered for a poor or incomplete response with persistent DMO. High retreatment burden, patient-related factors including adherence restrictions or disease-related characteristics (e.g. unresolved maculopathy) may impact clinical decisions on appropriate switch options and timings. Patients who are maintained stable with controlled diabetic maculopathy on longer dosing intervals of every 10 or 12 weeks are thought most likely to transition to a *pro re nata* (PRN, as needed) treatment plan with continued unchanged therapy. The Diabetic Retinopathy Clinical Research Network Protocol I study showed that the need for intravitreal anti-VEGF injections in DMO decreases annually over 5 years [45].

Ideally all suitable patients with previously treated DMO who are switched to faricimab would receive an initial monthly loading phase where possible, followed thereafter by a personalised T&E approach. Extension increments of 4 weeks are considered suitable, but there is flexibility for shorter adjustment increments at the discretion of the treating clinician.

Adjunctive macular laser or change to intravitreal corticosteroid treatment may be appropriate in eyes with suboptimal response to anti-VEGF agents.

In a landmark comparative effectiveness study of different anti-VEGF treatments for DMO, supplemental laser was administered if DMO persisted and was not improving starting at month 6; over 2 years, 41–65% of eyes received laser therapy at least once [46]. Macular laser can be effective for people who have visual impairment due to centre-involving DMO and have thinner retinas (CRT < 400 µm) and may delay the need for anti-VEGF treatment [47]. Either observation or macular laser may be considered for people with DMO and good vision (i.e. ≥79 letters) [47].

Vasoactive growth factors and inflammatory cytokines other than VEGF and Ang-2 contribute to the breakdown of the blood-retinal barrier and the onset of DMO [48]. Intravitreal corticosteroid implants dexamethasone (Ozurdex, AbbVie) and fluocinolone acetonide (Iluvien, Alimera Sciences) suppress inflammation and inhibit VEGF expression and are effective in reducing CRT and improving visual acuity in DMO; however, they are associated with a high risk of intraocular pressure elevation and the development or progression of cataract in phakic patients [49–52].

**Fig. 3:**
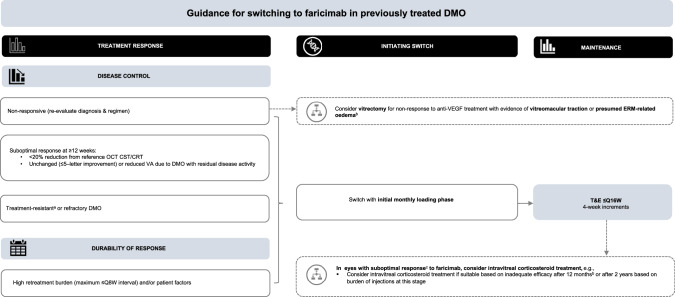
Guidance for switching to faricimab in previously treated DMO. ^a^Treatment-resistant DMO generally defined as persistent DMO, increased retinal thickness, or unchanged (≤5-letter improvement)/reduced VA due to DMO with residual disease activity, despite continued intravitreal anti-VEGF treatment over a 12-month period. ^b^[47]. ^c^Macular laser may be considered as adjunct therapy [47]. anti-VEGF anti-vascular endothelial growth factor, CRT central retinal thickness, CST central subfield thickness, DMO diabetic macular oedema, ERM epiretinal membrane, OCT optical coherence tomography, ≤Q8W every 4–8 weeks, Q16W every 16 weeks, T&E treat-and-extend, VA visual acuity.

It is recommended that clinicians consider intravitreal corticosteroid treatment if suitable based on inadequate response after 12 months or after 2 years based on burden of injections at this stage [47, 53]. Intravitreal corticosteroid treatment may also be considered in DMO eyes with contraindications to anti-VEGF [47]. Vitrectomy should be considered for DMO patients who do not respond to anti-VEGF treatment with evidence of vitreomacular traction or presumed ERM-related oedema [47]. Figure 3 provides an overview of guidance for switching to faricimab T&E in previously treated DMO.

Different treatment plans for each eye are feasible for patients with bilateral DMO (e.g. intravitreal corticosteroid treatment in one eye and anti-VEGF for the other).

The panel recommended non-ophthalmic physician review for all patients, especially those with uncontrolled diabetes and for patients with progressive retinopathy or resistant maculopathy, to address all treatable systemic risk factors. Routine access to support from diabetologists or general practitioners is recommended.

## General considerations when switching to faricimab among previously anti-VEGF-treated nAMD and DMO

### Bilateral involvement

For bilateral involvement, clinicians ideally use the same intravitreal anti-VEGF agent for both eyes; clinicians should therefore consider switching both eyes at the same time if possible when switching between these agents. If bilateral injections are planned during the same visit, clinicians should use a separate batch of medication for each eye or reschedule another appointment if separate lots are not available [54]. The safety and efficacy of faricimab administered in both eyes concurrently have not been studied [7].

### Patient counselling and consent

- Patients should be informed of potentially serious risks of intravitreal injection procedures as well as risks associated with intravitreal VEGF inhibitors [54]. Evidence suggests that treatment with faricimab has similar adverse events to aflibercept and ranibizumab (Lucentis, Novartis) [15–18, 55–57].
- Intraocular inflammation rates in phase III clinical trials with faricimab were low and comparable to aflibercept [19] (Table 1). Post marketing, rare cases of retinal vasculitis and/or retinal occlusive vasculitis have been reported with the use of faricimab and aflibercept [7, 58, 59]. Because these adverse reactions are reported voluntarily from a population of uncertain size, it is not always possible to reliably estimate their frequency, according to product label updates [7, 58, 59].
- When initiating intravitreal VEGF inhibitor treatment, caution is advised in nAMD patients with a large and/or high PED who may be at increased risk of retinal pigment epithelial (RPE) tear development [7, 60, 61]. Several reports suggest that eyes with a baseline PED height ≥600 µm have an increased risk of RPE tear following intravitreal anti-VEGF therapy [62, 63]. The frequency of RPE tear development through 2 years in the TENAYA and LUCERNE nAMD trials (pooled) was 2.9% with faricimab and 1.5% with aflibercept; most of these adverse events were reported as mild to moderate, without impact to vision and occurred during the loading phase [7].
- Documented patient consent obtained prior to first intravitreal injection procedure typically applies for a course of repeated anti-VEGF treatments. Professional guidance recommends that repeat written consent should be obtained if there is a change to the treatment plan or drug used, change in the clinical condition and/or change in perceived benefit-risk to the patient [34, 35].
- Some patients may need a period of continued monthly faricimab dosing after a loading phase to maximise treatment response.

### Post switch

- Patients who experience hypersensitivity or have worsening anatomic response following a treatment switch to faricimab could either be switched back to previous anti-VEGF or given a different anti-VEGF agent.

### Modelling of treatment regimens

- Clinical modelling of alternative treatments and regimens should ideally capture a range of related healthcare costs and benefits for the patient, the practice and the healthcare system overall, including: net acquisition, administration and monitoring cost of therapy; healthcare resource use and costs (e.g. OCT procedures, non-consultant led outpatient visits, nurse or technician-led monitoring, consultant-led reviews, post injection management and overall episode costs); capacity improvements over time, supported by modelling assumptions of injection frequencies through years 3–5; and impact on patients and their carers [64, 65].

## Discussion

This panel review provides practice considerations and recommendations for assessing response to intravitreal anti-VEGF therapy for nAMD and DMO and outlines potential options and approaches for switching to faricimab in eyes with suboptimal improvement or limited durability of response. Panel discussions on biomarkers of disease activity have concentrated on OCT-guided findings that can be routinely assessed in clinical practice. Lack of efficacy demonstrated by suboptimal anatomic or visual improvement and high treatment burden are the most common reasons for considering a change of treatment in patients with nAMD and DMO. Switch strategies guided by disease control and durability of response, and incorporating monthly loading doses or interval-matched initiation, may be integrated into current faricimab clinical pathways for treating patients with nAMD and DMO [33, 42].

Professional guidance recommends a treatment switch in patients with nAMD and DMO experiencing allergy or presumed tachyphylaxis and for practical reasons [34, 47]. Draft guidance from NICE on treating DMO recommends that clinicians should consider using macular laser as rescue treatment or changing anti-VEGF treatment if initial treatment alone does not stabilise (i.e. within 5 letters of visual acuity from baseline) or improve the patient’s vision after the loading phase [47]. A further review of treatment response should take place after 12 months and clinicians should consider intravitreal corticosteroid treatment if the response is suboptimal [47].

Clinical trial and early real-world data suggest that switching to faricimab may provide rapid anatomic improvement with stable or improved vision and with potential for extended treatment intervals in some patients with nAMD and DMO [66–70]. Across 21 National Health Service sites in the UK, early treatment patterns of faricimab among patients with nAMD and DMO previously treated with anti-VEGF confirm treatment interval extensions after the first 4 months with maintenance of vision [71, 72]. In a small retrospective cases series from a single UK tertiary care centre, faricimab was shown to be a safe and effective option for patients with treatment-resistant nAMD on aflibercept Q4W–Q6W, with clinical improvement on OCT observed in 80% of patients (*n* = 68) [73]. The authors noted that there was no evidence that switching with either 2 or 3 monthly loading injections had an independent effect on clinical outcomes [73].

This article focuses on anti-VEGF-experienced patients and treatment switch to dual Ang-2/VEGF pathway inhibition with faricimab as an option for suboptimal response or presumed acquired resistance to previous therapy. However, changing to a different anti-VEGF drug may also be a suitable option among patients who have an incomplete response or diminished response over time with previous anti-VEGF therapy [74, 75]. Moreover, reported outcomes of faricimab in previously anti-VEGF-treated patients are mostly limited to observational, non-controlled studies involving early data captured in routine practice, with no standardised measurements of visual acuity and variable clinician dosing approaches. An early positive anatomic and/or visual response following treatment switch may not necessarily translate to long-term patient response and more intensive treatment may provide additional clinical benefit in some eyes. Clinicians will continue to face heterogeneity in clinical response, including challenging or complex cases that do not respond satisfactorily to anti-VEGF treatments. Intravitreal corticosteroid implant treatment is a potentially effective option for patients with DMO who have an inadequate response to continued anti-VEGF therapy [47, 76, 77].

## Closing comments

To conclude, there was strong emphasis from panel members that disease control and frequency of injection should be at the forefront of clinician thinking when assessing treatment response and switch strategies in the management of nAMD and DMO. Emerging data suggest that dual Ang-2 and VEGF-A pathway inhibition with faricimab may offer potential for greater disease control with less frequent dosing over time compared with targeting the VEGF pathway alone [78]. Among patients who respond satisfactorily to faricimab, the treatment interval may potentially be extended, reducing burden and risks associated with frequent injections while maintaining or improving visual outcomes. Fewer injection and clinic monitoring visits provide meaningful benefits for patients and carers [79, 80]. Additionally, decreased injection frequency across a subset of patients may help to improve clinic capacity, thereby preventing treatment delays and potentially facilitating implementation of new or additional services [81].

Current clinician guidance on switch strategies may be further adapted as additional longer-term data and evidence emerge from multiple, ongoing real-world studies [71, 72, 78]. Future research will also add to current understanding of treatment patterns and patient outcomes utilising different switch strategies, including responses over time with and without a monthly initiation phase.

## Summary

### What was known before

- Some eyes fail to respond or only partially respond to multiple anti-VEGF treatments and frequent intravitreal injections are often needed to maintain efficacy.
- There is a need for standardised protocols defining treatment response and practical criteria for considering a treatment switch for patients who respond insufficiently to previous anti-VEGF therapy.

### What this study adds

- Clinician-led practice considerations and recommendations for assessing patient response to intravitreal anti-VEGF and potential options and approaches for switching to faricimab in eyes with suboptimal improvement or limited durability of response on previous anti-VEGF therapy.

## Data Availability

Consensus recommendations were developed based on objective expert opinion and clinical experience. Correspondence and requests for further information should be addressed to the corresponding author.
